# Recombinant cellobiohydrolase of *Myceliophthora thermophila*: characterization and applicability in cellulose saccharification

**DOI:** 10.1186/s13568-021-01311-8

**Published:** 2021-11-04

**Authors:** Anica Dadwal, Shilpa Sharma, Tulasi Satyanarayana

**Affiliations:** 1 Department of Biological Sciences & Engineering, Netaji Subhas Institute of Technology (University of Delhi), Azad Hind Fauj Marg, Sector-3 Dwarka, New Delhi, 110078 India; 2 Department of Biological Sciences & Engineering, Netaji Subhas University of Technology, Azad Hind Fauj Marg, Sector-3 Dwarka, New Delhi, 110078 India

**Keywords:** Thermostable cellulose, Recombinant cellobiohydrolase, Alkalistable, Solvent tolerance, Extremozyme, Cellulose saccharification

## Abstract

A codon optimized cellobiohydrolase (CBH) encoding synthetic gene of 1188 bp from a thermophilic mold *Myceliophthora thermophila* (*MtCel6A*) was cloned and heterologously expressed in *Escherichia coli* for the first time. In silico analysis suggested that MtCel6A is a GH6 CBH and belongs to CBHII family, which is structurally similar to Cel6A of *Humicola insolens*. The recombinant MtCel6A is expressed as active inclusion bodies, and the molecular mass of the purified enzyme is ~ 45 kDa. The rMtCel6A is active in a wide range of pH (4–12) and temperatures (40–100 °C) with optima at pH 10.0 and 60 °C. It exhibits T_1/2_ of 6.0 and 1.0 h at 60 and 90 °C, respectively. The rMtCel6A is an extremozyme with organic solvent, salt and alkali tolerance. The K_m_, V_max_, k_cat_ and k_cat_/K_m_ values of the enzyme are 3.2 mg mL^−1^, 222.2 μmol mg^−1^ min^−1^, 2492 s^−1^ and 778.7 s^−1^ mg^−1^ mL^−1^, respectively. The product analysis of rMtCel6A confirmed that it is an exoenzyme that acts from the non-reducing end of cellulose. The addition of rMtCel6A to the commercial cellulase mix (Cellic CTec2) led to 1.9-fold increase in saccharification of the pre-treated sugarcane bagasse. The rMtCel6A is a potential CBH that finds utility in industrial processes such as in bioethanol, paper pulp and textile industries.

**Supplementary Information:**

The online version contains supplementary material available at 10.1186/s13568-021-01311-8.

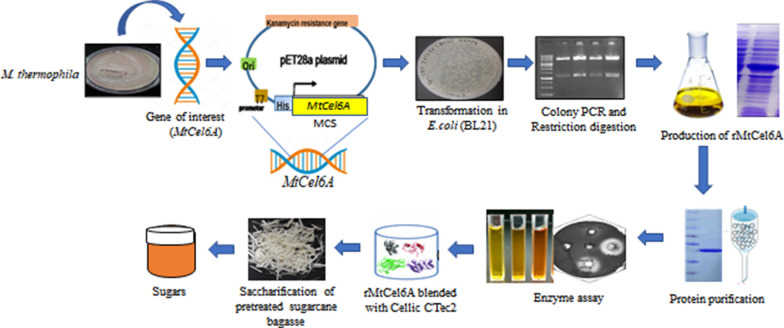

## Keypoints

- *MtCel6A has been cloned for the first time from* *Myceliophthora thermophila.*
- *It is a thermo-alkali stable enzyme with organic solvent and salt tolerance.*
- *This CBHII is compatible with Cellic CTec2 in improving cellulose saccharification.*

## Introduction

Lignocellulosic biomass is the abundant and renewable source of energy. In view of global warming and increasing demands for energy, the conversion of lignocellulosics to bioethanol is an attractive alternative (Liu et al. 2019). Lignocellulosics are predominantly composed of cellulose, hemicellulose and lignin. The enzymes that convert the complex and recalcitrant structure of cellulose are termed cellulases that includes three components cellobiohydrolase (CBH), endoglucanase (EGL) and β-glucosidase (BGL) (Himmel et al. 1999). Cellobiohydrolases (CBHs) are further classified as CBHI and CBHII based on their mode of action. CBHI acts on reducing ends, while CBHII acts on non-reducing ends of oligosaccharides of varied length to produce cellobiose. All three enzymes act synergistically to saccharify cellulose (Horn et al. 2012). The sugars generated from the conversion process are further fermented to ethanol utilizing yeast strains such as *Saccharomyces cerevisiae* (Kang et al. 2014). According to the CAZy (Carbohydrate Active Enzymes) database (http://www.cazy.org), the cellulases are classified into different GH families. The hydrolytic enzymes are indispensable for biomass valorization (Manisha 2017). The CBHs typically belong to GH5-GH7, GH9 and GH48 families. The cellulases are produced naturally by various fungal, bacterial and archaeal species (Obeng et al. 2017) and the cellulases produced by thermophilic microbes are typically thermostable.

Thermostable enzymes are of immense interest because of various attractive features they offer, including the presence of activity at elevated temperatures, lower risk of contamination, ability to withstand high pH and organic solvents (Dadwal et al. 2021; Liu et al. 2019). The thermostable cellulases are in great demand in various biotechnological industries including bioethanol production (Suresh et al. 2021; Turner et al. 2007). Over the years, several investigations have been carried out for evolving efficient thermostable cellulases through various genetic/protein engineering techniques (Dadwal et al. 2020). To fulfil the immense demand for the thermostable cellulases for industrial applications, heterologous expression has become the crucial technique that often significantly improves protein expression levels (Lambertz et al. 2014). The formation of inclusion bodies (IBs) is, however, regarded as one of the limitations in recombinant protein production in *Escherichia coli*. In the last few years, the concept of IBs being only inactive protein aggregates has changed and the concept of non-classical IBs emerged (Slouka et al. 2019). There are certain advantages associated with these IBs such as their easy isolation and purification process, resistance to proteolytic degradation, mechanical stability, overexpression, native-like secondary structure of protein in IBs, and the presence of biological activity (Singhvi et al. 2020). A few studies have reported a high yield and activity of the recombinant proteins produced in IBs in contrast to the recombinant proteins secreted under optimal conditions (Gundinger and Spadiut 2017; Jong et al. 2017).

*Myceliphthora thermophila* (syn. *Thermothelomyces thermophilus, Sporotrichum thermophile*) is a thermophilic mold that grows optimally at 45 °C (Morgenstern et al. 2012). This mold is a source of a series of glycoside hydrolases including cellulases. There are eight CBHs in the genome of this mold, but only two of them have been cloned and characterized (Gusakov et al. 2005; Kadowaki et al. 2018); both of them belong to the GH7 family. The investigations on GH6 cellobiohydrolases have not been reported often. However, a few GH6 cellulases have been explored, such as those of *Cellulosimicrobium funkei* CelL (Kim et al. 2016), *Thermobifida halotolerans* GH6 endoglucanase (Yin et al. 2015) and *Thermobifida fusca* Cel6A (Ali et al. 2015).

In this investigation, a GH6 cellobiohydrolase gene of *M. thermophila* (*MtCel6A*) was cloned, expressed and characterized for the first time. All fungal GH6 and GH7 CBHs are classified as CBHII and CBHI, respectively (Teter et al. 2014), and MtCel6A belongs to CBHII family that acts from the non-reducing ends of cellulose chains. The synthetic gene (*MtCel6A*) was heterologously expressed in *E. coli*. The recombinant enzyme is produced in the form of non-classical inclusion bodies (IBs), which is further solubilized, refolded, purified and characterized. The rMtCel6A is tolerant to various organic solvents and metal ions. It exhibits a good detergent and salt tolerance too. Moreover, we found that it efficiently degrades crystalline cellulosic biomass such as sugarcane bagasse, and the hydrolysis product mainly is cellobiose. Furthermore, the synergistic action of rMtCel6A with a commercial cellulase cocktail (Cellic CTec2) on the pretreated sugarcane bagasse has also been confirmed. This study suggests that the recombinant MtCel6A is a good biocatalyst of commercial value in the various biotechnological processes where alkaline conditions prevail in the saccharification of alkali-pretreated lignocellulosic biomass.

## Materials & methods

### Microbial strains, plasmids and reagents

*E. coli* DH5α and *E. coli* BL21 (DE3) were used for plasmid replication and expression in host cells, respectively. Both these strains were procured from Invitrogen (CA, USA). The Luria–Bertani (LB) medium purchased from Hi-Media (Mumbai, India) was used for maintaining the strains. The pET-28a vector (Invitrogen, USA) was used as a cloning and expression vector. Restriction enzymes and the DNA ligation kit were procured from New England Biolabs (MA, USA). For determining the enzyme activity, all the substrates were bought from Sigma (USA). Cellic CTec2 was supplied by Novozymes (Bagsværd, Denmark). All other reagents used were of analytical grade and purchased from Sigma (St. Louis, USA), Merck (Darmstadt, Germany), Fisher Scientific (Massachusetts, USA), CDH (India) and SRL (India) and Hi-Media (Mumbai, India). Sugarcane bagasse used in the study was collected from a local juice shop.

### In silico analysis of MtCel6A

The 3D protein model of MtCel6A was constructed using Swiss-Model, (https://swissmodel.expasy.org/) an online tool of Expasy. The protein sequence similar to MtCel6A was retrieved using BLAST-P (Basic Local Alignment Search Tool for Proteins) program (https://blast.ncbi.nlm.nih.gov/Blast.cgi). Multiple sequence alignments were made using ESPript 3.0 (https://espript.ibcp.fr/).

### Construction of MtCel6A expression vector

The *Cel6A* gene of *M. thermophila s*yn. *Thermothelomyces thermophilus* (accession number AEO59280.1) with Codon Adaptation Index (CAI) of 0.67 was codon optimized to CAI of 0.88 for expression in *E. coli* (GenBank accession no. MZ826702). The synthetic codon optimized gene was synthesized and supplied by Biomatik (Canada). Gene-specific primers (FP: 5′ GGCGGATCCATGAAATTTGTTCAGAGCGC 3′ and RP: 5′ CGCAAGCTCTTAAAAGCTCGGATTGGC 3′) were used for amplifying *MtCel6A* (1188 bp). The double digestion of amplified *MtCel6A* and pET-28a was carried out using *Bam*HI and *Hin*dIII to generate sticky ends and were ligated using ligation kit. The ligated product was further transformed into *E. coli* DH5α. Kanamycin resistant *E. coli* colonies were selected on LB supplemented with 50 µg mL^−1^ kanamycin (GoldBio, USA).The positive colonies harbouring *rMtCel6A*-pET-28a were confirmed by colony PCR and double digestion of the plasmid with *Bam*HI and *Hin*dIII. The pET-28a plasmid containing *MtCel6A* was transformed into chemically competent *E. coli* BL21 (DE3). The recombinant MtCel6A was produced from positive clones.

### Production of rMtCel6A

For the expression of recombinant MtCel6A, *E. coli* BL21 (DE3) harbouring pET-28a-*MtCel6A* was cultivated in 1 L LB medium (supplemented with 50 µg mL^−1^ kanamycin) and grown at 37 °C to the optical density (λ—600 nm) of 0.5. The gene expression was induced by 0.25 mM isopropyl β-D-1-thiogalactopyranoside (IPTG) at 30 °C. The cells were harvested after 2 h by centrifugation (8,000 rpm for 20 min at 4 °C). The resultant cell pellet was washed with Tris–HCl buffer (pH 8.0) and resuspended in 10 mL lysis buffer [50 mM Tris–HCl (pH 8.0), 300 mM sodium chloride (NaCl), 1% triton X-100, 1 mM phenylmethylsulfonyl fluoride (PMSF)]. The cell suspension was sonicated to disrupt the cells following sonication cycles of 5 s on and 5 s off for 20 min. The lysate was centrifuged (12,000 rpm for 30 min at 4 °C) and the pellet was stored at -20 °C for isolating IBs.

### Isolation, solubilization and refolding of rMtCel6A from inclusion bodies

The isolation and purification of IBs from cell pellet was performed according to Palmer and Wingfield (2012). For solubilization of IBs, six solubilization buffers were used: [Buffer 1 (B1): Tris buffer (pH 10.0), Buffer 2 (B2): Tris buffer (pH 10.0) + 5% dimethylsulfoxide (DMSO), Buffer 3 (B3): Tris buffer (pH 11.0), Buffer 4 (B4): Tris buffer (pH 11.0) + 5% DMSO, Buffer 5 (B5): Tris buffer (pH 12.5), Buffer 6 (B6): Tris buffer (pH 12.5) + 5% DMSO]. After solubilization, the samples were centrifuged (12,000 rpm for 15 min at 4 ºC) and the supernatants were loaded on 12% SDS PAGE to analyse the protein. The solubilization was assessed in terms of turbidity and total protein content by recording absorbance at 600 and 280 nm, respectively. Solubilization (%) was calculated by solubilizing IBs (1 mg mL^−1^) in different buffers and determining protein content in supernatant by Lowry’s method (Waterborg 2009). The solubilized rMtCel6A IBs were refolded by pulsatile renaturation process using refolding buffer [50 mM Tris–HCl (pH 10.0), 10% glycerol, 5 mM dithiothreitol (DTT), 1 mM PMSF] in 1:9 ratio at 4 °C with constant agitation. The refolded rMtCel6A protein was further concentrated using Amicon centrifugal filters with 30 kDa molecular weight cut-off.

### Purification of rMtCel6A

After refolding, the purification of rMtCel6A was performed from IBs according to the manufacturer’s instructions (Qiagen, Germany). The proteins were eluted with an increasing gradient of imidazole concentration (50–150 mM). The purified protein was stored at 4 ºC and the purity of the enzyme was checked by electrophoresis on 12% SDS-PAGE. Protein concentration of the purified rMtCel6A was determined by the Lowry’s method with bovine serum albumin (BSA) as the standard.

### Zymogram analysis

For detection of CBH activity of rMtCel6A, zymogram analysis was performed. The purified rMtCel6A was run on a 12% (w/v) Native-PAGE. At the end of the electrophoresis, the gel was rinsed and equilibrated with buffer (pH 10.0). Subsequently, the gel was overlaid on 2.5 µM 4-methylumbelliferyl β-D-cellobioside (4-MUC) (a fluorescent substrate) agar plate and incubated in dark for 30 min at 50 °C, followed by visualizing of the plate under UV light for detecting fluorescence due to the release of 4-methylumbelliferone.

### Qualitative and quantitative cellobiohydrolase assay

For qualitative assay of CBH, 2.5 µM of 4-MUC was used along with 1% agarose in buffer (pH 10.0) and poured into Petridish. After solidifying, the wells were punched and various fractions of rMtCel6A (200 µl) were dispensed into these wells. The Petridish was incubated in dark for 60 min at 50 °C. The fluorescence was observed under UV light.

For quantitative detection of CBH activity, the reaction mixture containing 0.5 mL substrate [0.5% (w/v) Avicel PH-101 prepared in 0.1 M buffer (pH 10.0) and 0.5 mL appropriately diluted enzyme (rMtCel6A) was incubated at 60 °C for 30 min. After completion of hydrolysis, 1 mL of dinitrosalicylic acid (DNS) reagent was added to the reaction mixture and incubated at 100 °C for 10 min. The reducing sugars liberated were quantitated by reading absorbance at 540 nm (Miller 1959). One unit of cellobiohydrolase is defined as the amount of enzyme that liberates 1 μmol of reducing sugars as glucose min^−1^ under the assay conditions.

### Circular dichroism spectroscopy

Far-UV circular dichroism (CD) spectra of purified rMtCel6A in buffer were recorded in the range 190–260 nm in a Jasco J-815 Spectropolarimeter (Jasco International Co.) using a 1 mm quartz cuvette at scanning speed, band width, and digital integration time (D.I.T) of 100 nm min^−1^, 1 nm, and 1 s, respectively. A total of 3 spectra were collected, averaged and corrected by subtracting the blank. The results are expressed as CD signal (measured ellipticity) in mdeg (millidegrees).

### Substrate specificity of rMtCel6A

To study substrate specificity of rMtCel6A, different substrates [carboxymethylcellulose (CMC), avicel, filter paper, cotton, microcrystalline cellulose (MCC) and birchwood xylan] were used. Enzyme activity was determined using DNS reagent (Miller 1959).

### Biochemical characterization of purified rMtCel6A

#### Determination of optimum pH and temperature

The purified rMtCel6A and 0.5% (w/v) avicel was prepared in buffers of varying pH (0.1 M, pH 4.0–10.0). The enzyme reaction was carried out to determine the pH optimum. The optimum temperature for rMtCel6A was derived from the enzyme assays performed at varied temperatures (40–100 °C, pH 10.0) for 30 min with 0.5% avicel. The reducing sugars liberated were quantitated.

#### Determination of thermostability

The enzyme was incubated at various temperatures (60–100 ºC) for varied time intervals to determine the thermostability. The aliquots were taken at the desired time intervals and kept in ice for an hour, followed by enzyme assay at 60 °C.

#### Enzyme kinetics of rMtCel6A

Kinetic parameters were evaluated from purified rMtCel6A by incubating the enzyme at 60 °C with different concentrations of avicel (5–30 mg mL^−1^) in 0.1 M of sodium carbonate-bicarbonate buffer (pH 10.0). A Lineweaver–Burk graph was plotted to calculate Michaelis constant (K_m_) and the maximum enzyme action velocity (V_max_) values. The turnover number (k_cat_) values were calculated from V_max_ and the molar enzyme concentration used in the reaction. The catalytic efficiency was calculated by dividing k_cat_ with K_m_ value.

#### Effect of metal ions, organic solvents, detergents and enzyme inhibitors on the activity of rMtCel6A

The purified recombinant enzyme was incubated with avicel in the presence of different metal ions (5 mM of Na^+^, Ca^2+^, Co^2+^, K^+^, Mg^2+^, Mn^2+^, Fe^2+^, Cu^2+^, Zn^2+^, NH_4_^+^), organic solvents (acetone, butanol, chloroform, ethanol, ethyl acetate, hexane, isoamyl-alcohol, isopropanol, liquor ammonia and methanol) at 10% (v/v), detergents such as sodium dodecyl sulphate (SDS), tween-80, triton X-100, cetyltrimethylammonium bromide (CTAB), and polyethylene glycol (PEG) at 0.5% (w/v) and different modulators (5.0 mM concentration) such as iodoacetamide (IA), diethylpyrocarbonate (DEPC), dithiothreitol (DTT), ethyl-3-(3-dimethyl aminopropyl) carbodiimide (EDAC), ethylenediaminetetraacetic acid (EDTA), N-bromosuccinimide (NBS), PMSF, Woodward’s reagent K (WRK). The activity of the enzyme under standard assay conditions without any additives was considered as the control with 100% enzyme activity.

#### Effect of NaCl

The purified recombinant enzyme was incubated with avicel in the presence of varying concentrations of NaCl (0.5—3 M) at 60 °C for 30 min. The enzyme activity was quantified in each sample.

### Utility of rMtCel6A in saccharification of pre-treated sugarcane bagasse

The sugarcane bagasse was washed thoroughly with tap water and later with distilled water. The residue was cut into 1–2 inch pieces and pre-treated with sodium sulphite (Na_2_SO_3_). After pretreatment, it was further washed to neutral pH, dried and ground to 30 mesh size for hydrolysis. The biomass loading of 4% (w/v) with 50 units of pure rMtCel6A were added to 10 mL buffer (pH 10.0) followed by saccharification at 60 °C, 150 rpm for 6 h. The rMtCel6A was also used in various ratios with appropriately diluted commercial enzyme cocktail (Cellic CTec2) in order to study their synergistic effect. The reducing sugars thus liberated were quantitated in the hydrolysates using DNS reagent (Miller 1959). The products of enzymatic hydrolysis were examined by spotting the supernatant on pre-coated silica gel thin layer chromatographic (TLC) plates (60 F254, Merck). The mobile phase of butanol: acetone:/water (5:3:2) was used. The products were detected by spraying the plate with diphenylaniline reagent followed by air drying and exposure to 100 °C for 5 min (Anderson et al. 2000).

The % saccharification and degree of synergy (DOS) were calculated using the following formulae (1) and (2), respectively:

1 $${\text{Saccharification}} \left( \% \right) = \frac{{{\text{Amount of reducing sugar liberated }}\left( {{\text{mg}}} \right) \times { }0.9{ } \times { }100}}{{{\text{total cellulose content in the biomass }}\left( {{\text{mg}}} \right)}}$$

2 $${\text{DOS}} = { }\frac{{\text{total sugars released from enzyme mix}}}{{\text{total sugars released from individual enzymes}}}$$

DOS > 1.0 and DOS ≤ 1.0 depict the status of synergy and no synergy/competition, respectively among the enzymes tested.

All experiments have been performed in triplicate, and the observations are presented as average values with standard deviation.

## Results

### 3D model and catalytic amino acid residue of MtCel6A

**Fig. 1:**
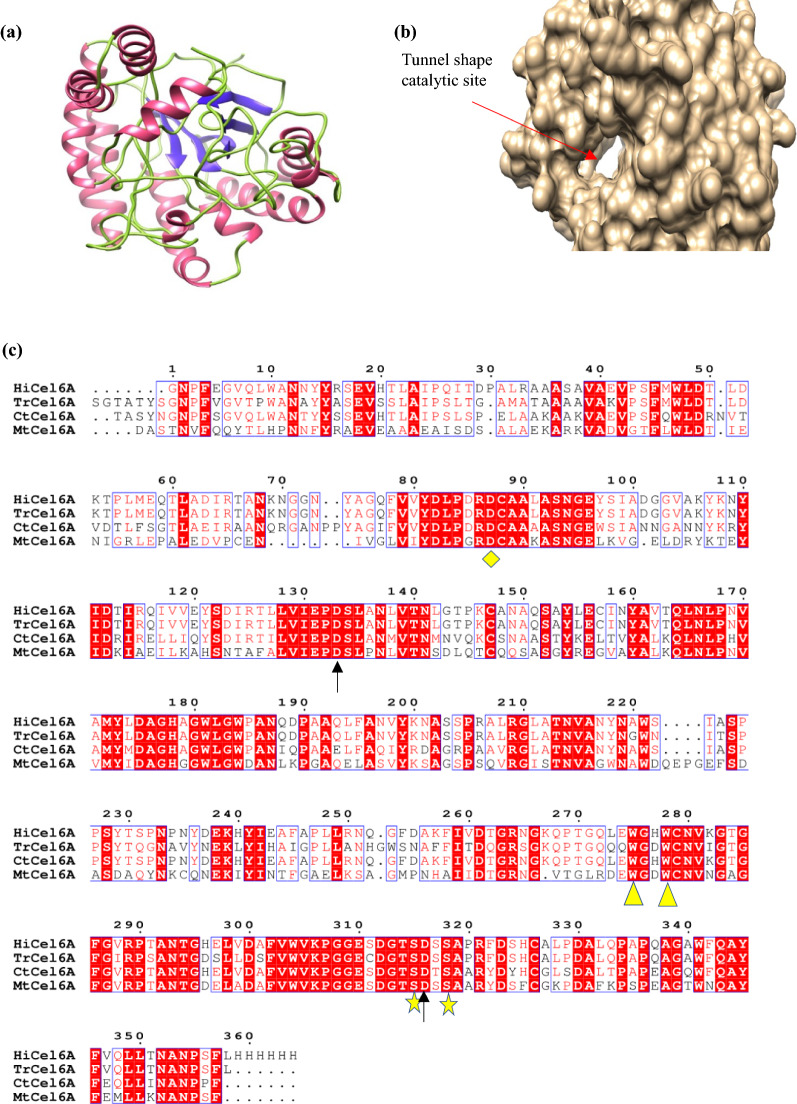
**a** 3D model of MtCel6A; where α-helix, β-sheets and loops are highlighted in purple, blue and green, respectively. **b** 3D structure showing tunnel shape catalytic site. **c** Multiple sequence alignment of MtCel6A showing conserved critical amino acid residues; Asp 128 (catalytic acid) and Asp 314 (catalytic base) are marked with arrows, and other key residues (described in text) are marked with yellow diamond, triangle, and star

BLAST-P analysis revealed that MtCel6A belongs to GH6 family that exhibits similarity with the GH6 CBHs of *Trichoderma reesei* (51%), *Humicola insolens* (53.7%) and *Chaetomium thermophilum* (52.5%). The 3D model of MtCel6A generated using Cel6A of *H. insolens* (PDB ID: 4I5R) as template that displayed maximum (54.18%) sequence identity. The 3D-model comprises 7-stranded (β/α) barrel fold and a tunnel shape catalytic site [Fig. 1a, b].

From multiple sequence alignment [Fig. 1c], various conserved regions were identified. Asp 128 identified as a catalytic acid that act as proton donor and Asp 314 may be a catalytic base. The amino acid residue Asp 83 is suggested to play a role in protonation of Asp 128 (Rouvinen et al. 1990). A few conserved short peptides, highlighted in red box in Fig. 1c, have also been identified.

### Production of rMtCel6A

**Fig. 2:**
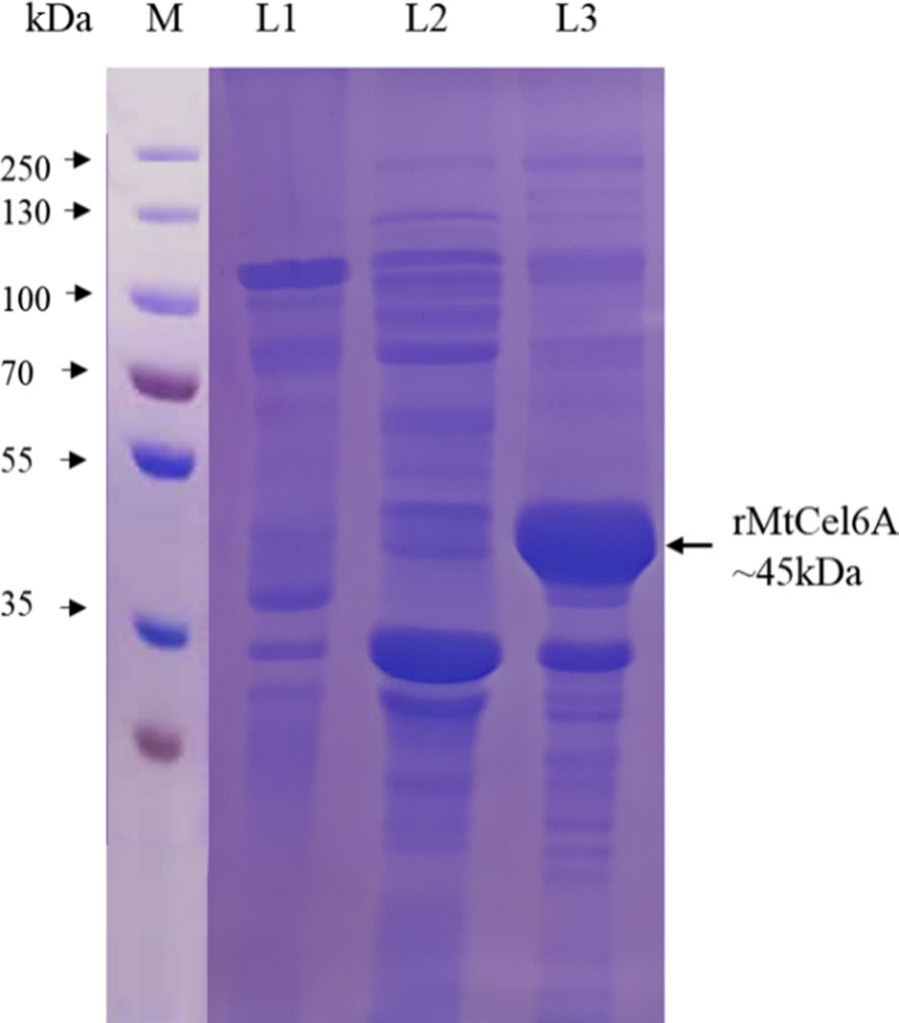
Production of rMtCel6A under optimum conditions. The protein samples loaded on 12% SDS PAGE; M: protein marker; L1: Empty vector (without construct) L2: rMtCel6A (supernatant); L3: rMtCel6A (pellet). [Protein ladder (PageRuler Plus prestained) was procured from Thermo Scientific]

The *MtCel6A* (synthesized gene) was amplified from the synthetic plasmid. Further, the cloning of synthetic *MtCel6A* in pET-28a vector was confirmed by double digestion of the vector. During the recombinant enzyme expression in *E. coli*, the enzyme was not detected in the soluble fraction obtained following *E. coli* cell lysis. The rMtCel6A accumulates in the bacterial IBs (Fig. 2) which were solubilized and refolded in order to obtain active enzyme. The enzyme production attained was about 6000 U L^−1^.

### Solubilization and refolding of rMtCel6A inclusion bodies

**Fig. 3:**
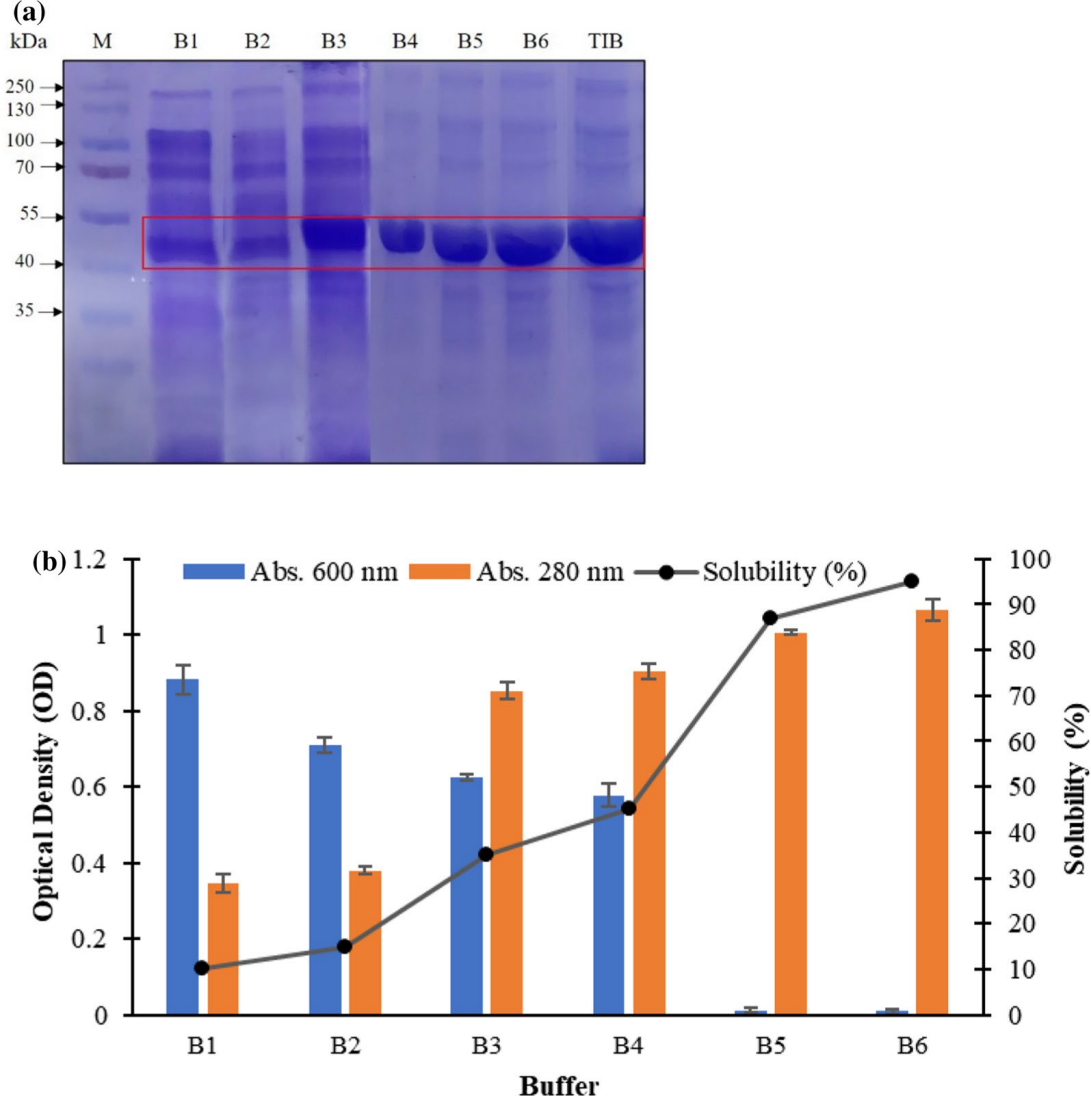
Solubilization of rMtCel6A purified IBs using different solubilization buffers **a** SDS gel of solubilized IBs; M: protein marker; B1: Buffer 1, B2: Buffer 2, B3: Buffer 3, B4: Buffer 4, B5: Buffer 5, B6: Buffer 6 (composition as described in text), TIB: Total Inclusion Bodies [Protein ladder (PageRuler prestained) was procured from Thermo Scientific] **b** Graph showing % solubility and absorbance of solubilized IBs at 600 nm and 280 nm showing turbidity, protein content, respectively

The observations suggested that buffer B6 [pH (12.5) + 5% DMSO] facilitates the maximum solubilization of rMtCel6A IBs. Thus, B6 was selected based on high solubilization and high protein content attained in the supernatant [Fig. 3a, b].

### Purification of the rMtCel6A

**Fig. 4:**
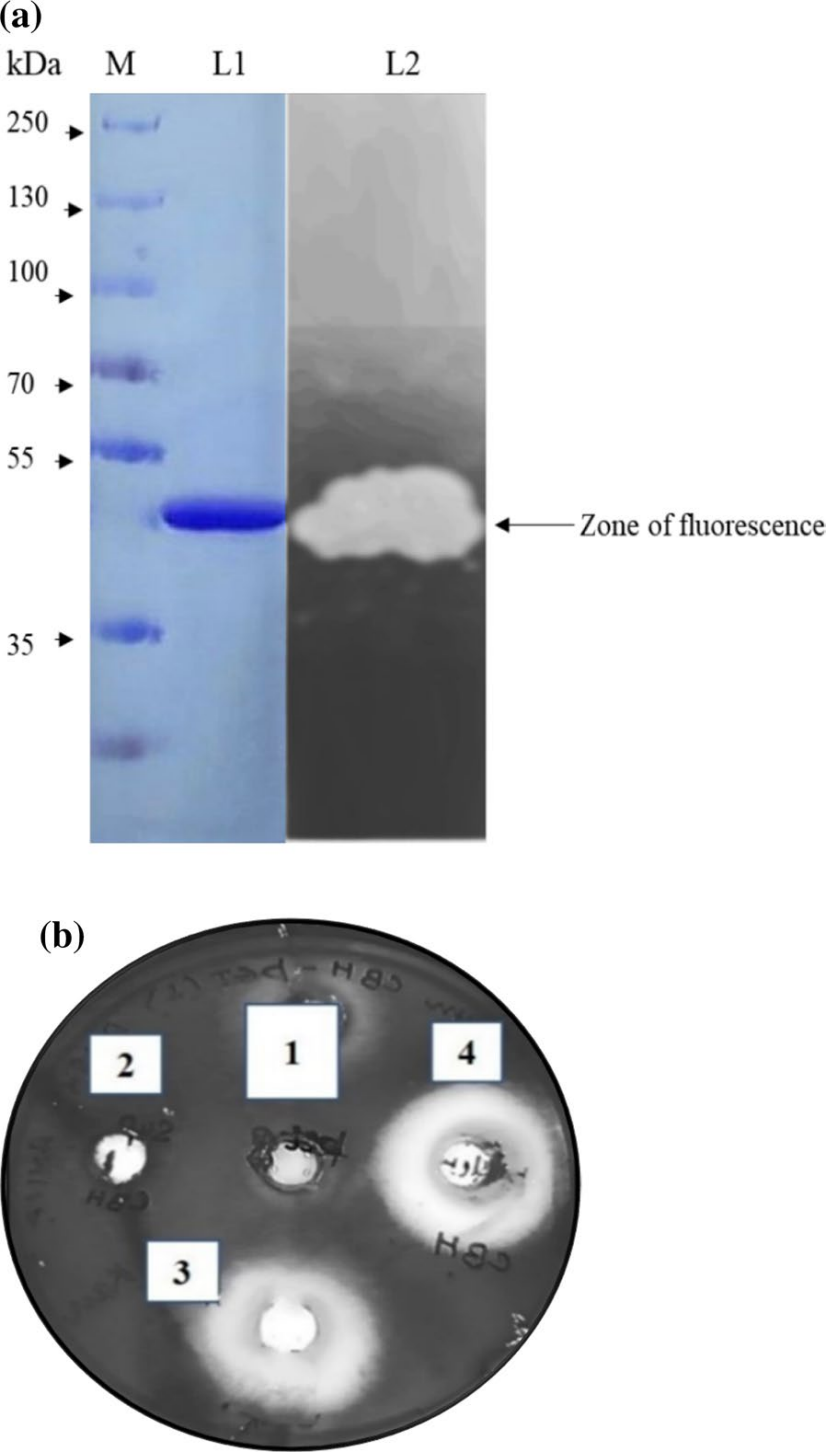
**a** Purification and zymogram of rMtCel6A. M: protein marker, L1: purified rMtCel6A, L3: zymogram showing fluorescence [Protein ladder (PageRuler Plus prestained) was procured from Thermo Scientific] **b** Qualitative cellobiohydrolase assay of various fractions of rMtCel6A using 4-MUC as substrate, where well 1: negative control (pH 10 buffer only), well 2: supernatant fraction obtained after lysis of rMtCel6A; well 3: rMtCel6A pure IBs (unfolded); well 4: solubilized and refolded IBs of rMtCel6A

The predicted mass of MtCel6A is 42 kDa as calculated using Protparam tool of Expasy (https://web.expasy.org/protparam/). However, the rMtCel6A was purified to homogeneity from solubilized and refolded IBs, the enzyme was of ~ 45 kDa protein at twofold purification, 57% yield and specific activity of 172 U mg^−1^ (Table 1). A little higher mass of the purified enzyme was recorded. By affinity chromatography, the protein was eluted at 120 mM imidazole and the purity was confirmed by SDS-PAGE [Fig. 4 (a)]. The zymogram analysis of the purified rMtCel6A exhibited fluorescence under UV light. [Fig. 4 (a)].

**Table 1:** Purification profile of rMtCel6A

Fraction of MtCel6A	Total enzyme activity (U)	Total protein concentration (mg)	Specific enzyme activity (U mg^−1^)	Yield (%)	Purification (fold)
Pure IBs	3000	35	85	100	1
Soluble IBs	2600	21	124	87	1.45
Refolded IBs	1920	12.5	153	64	1.8
His-Tag column purified	1720	10	172	57	2.0

### Enzyme assays

The enzyme activity in various fractions of rMtCel6A was assessed qualitatively [Fig. 4b]. The high fluorescence was observed in well no. 4 which contained refolded IBs, while well no. 3 contained unfolded IBs which displayed comparatively low fluorescence. As depicted in Table 1, the refolded rMtCel6A displayed 1.23-fold enhanced enzyme activity in comparison with the unfolded enzyme. The purified enzyme exhibited specific activity of 85 U mg^−1^.

### Secondary structure of the enzyme

The secondary structure of refolded rMtCel6A comprised 26.5% α-helix, 5.9% β-strands, 31.9% turns and 35.7% random coils, while the secondary structure composition of unfolded rMtCel6A IBs displayed 22.9% α-helix, 26.1% turns and 51% random coils, with no detectable β-strands (Additional file 1: Fig. S1).

### Substrate specificity

The substrate specificity of rMtCel6A on various substrates is shown in Table 2. It is evident that MtCel6A is highly efficient on the microcrystalline cellulose depolymerization of avicel as compared to crystalline cellulose and filter paper with specific activity values 172, 160 and 146 U mg^−1^, respectively with > 85% relative activity on all three substrates. However, it exhibits a low relative activity on cotton and wheat bran.

**Table 2:** Substrate specificities of rMtCel6A

Substrate	Specific activity (U mg^−1^)	Relative enzyme activity (%)
Avicel PH-101	172	100
Crystalline cellulose	160	93
Filter paper	146	85
Cotton	112	65
Wheat bran	103	60
CMC	ND	ND
Birchwood xylan	ND	ND

*ND* Not detected

### Biochemical characteristics of rMtCel6A

#### Optimum pH and temperature, and thermal stability

**Fig. 5:**
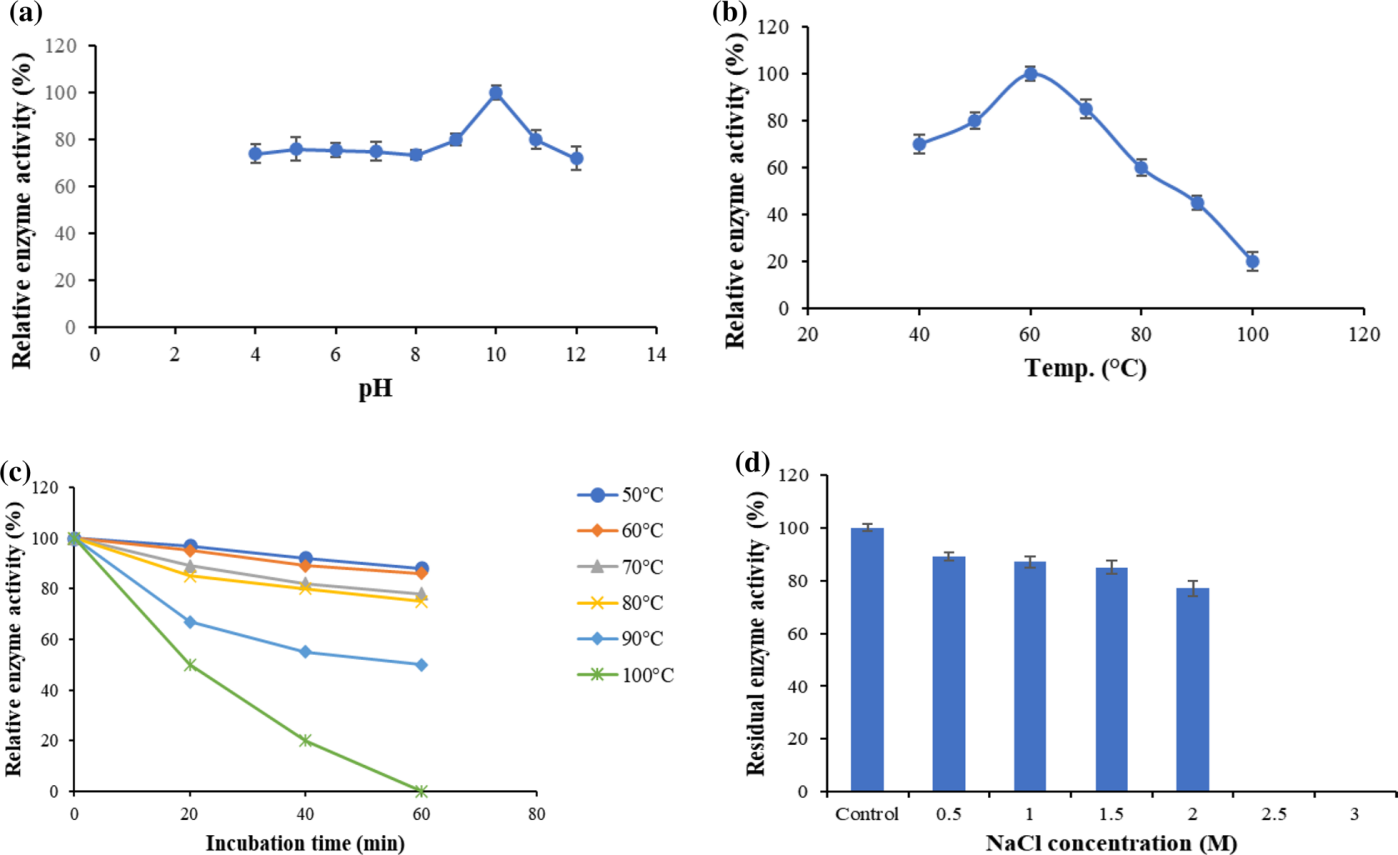
Effect of **a** pH, and **b** temperature on rMtCel6A activity, **c** Thermostability of rMtCel6A, **d** Effect of NaCl on rMtCel6A activity

The rMtCel6A is active in a broad range of pH (5.0 to 12.0) with optimum of 10.0 [Fig. 5a]. It is a thermostable enzyme with temperature optima of 60 °C [Fig. 5b]. The enzyme retained over 60% activity between 50–80 °C. At 90 and 100 °C, rMtCel6A retained 45 and 20% activity, respectively. The rMtCel6A displays activity at varied temperatures [Fig. 5c] and it exhibits T_1/2_ of 7, 6, 4, 3.5, 1.0 and 0.2 h at 50, 60, 70, 80, 90 and 100 °C, respectively.

#### Enzyme kinetics

rMtCel6A exhibited a K_m_ of 3.2 mg mL^−1^, V_max_ of 222.2 μM mg^−1^ min^−1^, k_cat_ of 2491.8 s^−1^ and catalytic efficiency (k_cat_/K_m_) of 778.68 s^−1^ mg^−1^ mL^−1^, when avicel was used as the substrate.

#### Effect of various modulators

In the presence of most of the metal ions (Na^+^, Ca^2+^, K^+^, Mg^2+^, Fe^2+^, Zn^2+^, NH_4_^+^) tested, the rMtCel6A retains > 80% activity. While three metal ions (Co^2+^, Mn^2+^ and Cu^2+^) exerted stimulatory effect with 113, 114 and 110% of relative activity (Table 3). The presence of solvents such as acetone, ethanol, liquor ammonia, isoamyl-alcohol and hexane at 10% concentration stimulated rMtCel6A enzyme activity. More than 90% of enzyme activity was retained in the presence of chloroform and isopropanol, while the enzyme was completely denatured in the presence of ethyl-acetate. Butanol and methanol displayed no observable effect on rMtCel6A activity (Table 3). The enzyme is surfactant/detergent tolerant retaining > 75% of residual enzyme activity in the presence of surfactants like SDS, triton X-100, CTAB and PEG (Table 3). The presence of tween-80, however, displayed inhibition (33%). The effect of cationic surfactant CTAB is unfavourable with 78% residual enzyme activity in comparison with anionic surfactant (SDS), where residual enzyme activity is 86%. In the presence of WRK, EDAC and NBS, the enzyme activity was inhibited to a varied extent (Table 3).

**Table 3:** Effect of various additives on rMtCel6A activity

Additives	Residual rMtCel6A activity (%)	Additives	Residual rMtCel6A activity (%)
Control	100 ± 1.09	Control	100 ± 1.09
Metal ions	5 mM	Surfactants	0.5%
Na^+^ Ca^2+^ Co^2+^ K^+^ Mg^2+^ Mn^2+^ Fe^2+^ Cu^2+^ Zn^2+^ NH^+^	90 ± 1.20 88 ± 0.90 113 ± 1.24 94 ± 1.31 97 ± 0.90 114 ± 1.40 84 ± 1.52 110 ± 0.79 81 ± 0.99 89 ± 2.01	SDS Tween-80 Triton X-100 CTAB PEG	86 ± 2.09 67 ± 1.97 91 ± 1.76 78 ± 1.39 80 ± 1.80
Organic solvents	10%	Modulators	5 mM
Acetone Butanol Chloroform Ethanol Ethyl acetate Hexane Isoamyl-alcohol Iso-propanol Liquor ammonia Methanol	137 ± 1.67 100 ± 1.73 94 ± 1.70 103 ± 0.96 ND 118 ± 1.29 133 ± 1.87 98 ± 1.57 120 ± 1.26 100 ± 1.05	IA DEPC DTT EDAC EDTA NBS PMSF WRK	50 ± 1.80 70 ± 1.69 60 ± 0.97 40 ± 1.54 62 ± 1.04 45 ± 1.45 75 ± 1.28 25 ± 2.01

*ND* Not detected

#### Salt tolerance

The rMtCel6A was active in the presence of 1–2 M NaCl, and it declined on further increasing NaCl concentration and complete loss of enzyme activity was observed at 2.5 and 3 M NaCl. The enzyme retained 89% activity at 0.5 M NaCl, which declined to 77% at 2 M NaCl [Fig. 5d].

### Applicability of rMtCel6A in saccharification of pre-treated sugarcane bagasse

#### End-products analysis and synergistic effect of MtCel6A with commercial cellulase cocktail (Cellic CTec2)

**Fig. 6:**
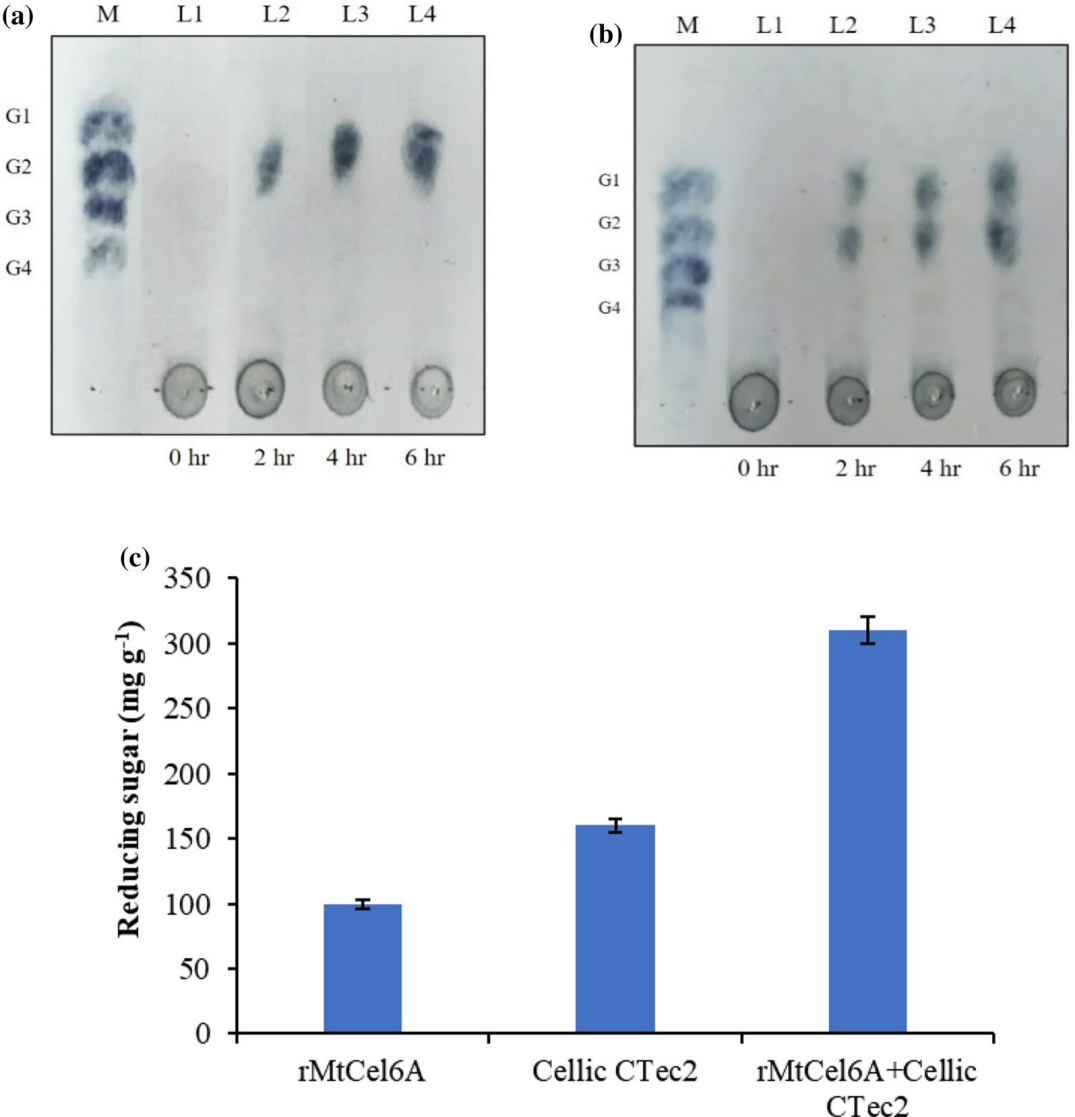
End product analysis of sugarcane bagasse hydrolysis by TLC; where M shows sugar standards and L1-L4 show the hydrolytic products obtained in regular time intervals **a** rMtCel6A alone **b** rMtCel6A + Cellic CTec2 **c** Reducing sugars liberated after hydrolysis of sugarcane bagasse utilizing Cellic CTec2, rMtCel6A and their combination

The final product of sugarcane bagasse hydrolysis was mainly a disaccharide, cellobiose [Fig. 6a]. The amount of oligosaccharide products released from the biomass gradually increased in a time dependent manner. The release of sugars was observed in the hydrolysates, when rMtCel6A was used in combination with cellulosic enzyme cocktail (Cellic CTec2) [Fig. 6b]. After 6 h of hydrolysis, a marked increase in reducing sugar yield was attained in the hydrolysates, when the blend of both enzymes were used in comparison with those attained by Cellic CTec2 and rMtCel6A alone [Fig. 6c]. The degree of synergy between rMtCel6A and Cellic CTec2 was 1.2 with a marked increase in relative saccharification to 194% (Table 4).

**Table 4:** Saccharification profile of rMtCel6A in combination with Cellic CTec2

Enzymes	Reducing sugar liberated (mg g^−1^)	% Saccharification	% Relative saccharification	Degree of synergy (DOS)
Cellic CTec2 (Control)	160 ± 0.8	36	100	NA
rMtCel6A	100 ± 1.2	22.5	62.5	NA
rMtCel6A + Cellic CTec2	310 ± 1.8	70	194	1.2

*NA* Not applicable

## Discussion

A recombinant MtCel6A (GH6) cellobiohydrolase of *M. thermophila* has been heterologously expressed and characterized for the first time. The 3D model of MtCel6A suggests to contain 7-stranded (β/α) barrel fold and a tunnel shape catalytic site, which is a key structural feature of Cel6A family of enzymes (Varrot et al. 1999). Multiple sequence alignment suggests that Asp residues are critical in enzyme catalysis. Earlier investigations revealed that the identification of catalytic base is less certain in Cel6A. Asp 128 (proton donor) of MtCel6A corresponds to Asp 226 and Asp 221 of Cel6A of *H. insolens* and *T. reesei,* respectively (Varrot et al. 1999; Rouvinen et al. 1990). Trp residues 273 and 276 are known to interact with the glucose sugar rings (Rouvinen et al. 1990; Varrot et al. 1999). Two conserved cysteine residues (Cys 313 and Cys 316) have been considered to stabilize the loops involved in the formation of active site tunnel (Rouvinen et al. 1990). Other conserved residues have also been identified, which could be critical in substrate binding, catalysis and thermostability.

The rMtCel6A is produced in the form of IBs in *E. coli*. These IBs are solubilized using mild solubilizing agents that help in restoration of the native like secondary structure of the protein and in the recovery of bioactive protein (Singh et al. 2015). The mild solubilizing agents either have a high pH or high salinity, and are devoid of any denaturants. The presence of enzyme activity in unfolded protein depicts that rMtCel6A is not completely inactive even in the form of IBs. It has been reported earlier that some non-classical IBs are active as they possesses a large amount of properly folded target protein (Singhvi et al. 2020). Therefore, rMtCel6A is thus expressed in the form of non-classical IBs in *E. coli*. Refolding, however, led to an enhancement in enzyme activity.

The prediction of rMtCel6A secondary structure based on the CD spectra is consistent with the previous reports that Cel6A of *Trichoderma reesei* displays an approximately similar proportion of 35.8% α-helices and 8.7% β-strands. In contrast, a cellobiohydrolase of *A. fumigatus* strain (AfCel6A) contained 27% α-helix and 7.7% β-strands (Bernardi et al. 2021). The presence of intact β-strands and a decline in random coils in the refolded IBs of MtCel6A confirms restoration of its secondary structure during the refolding process. The CD spectra of rMtCel6A showed a positive peak from 200–210 nm and a broad negative peak near 210 nm which extended till 240 nm, suggesting the dominance of α-helices in the secondary structure of MtCel6A.

The crystalline cellulose is the substrate preference of CBHs and this enzyme activity is a vital step in cellulose degradation (Lynd et al. 2002). In contrast, CMC is a soluble substrate and is usually not hydrolyzed by CBHs (Tonozuka et al. 2014). The observations recorded in this investigation are in agreement with this feature; no activity of rMtCel6A was detected on CMC. This could be explained by the fact that the catalytic site of MtCel6A is typically enclosed by a tunnel shape structure which does not allow soluble substrate like CMC to access the active site (Varrot et al. 1999). Thus, observations further suggest that this is a processive enzyme containing highest exo-catalytic activity on crystalline cellulose. The specific activity of this enzyme on avicel is higher than those reported for other CBHs (Dos Santos et al. 2020; Gu et al. 2019) making it an efficient Cbh. The rMtCel6A specifically attacks β-1,4-glycosidic bonds from the non-reducing ends of the crystalline cellulose and is remarkable in hydrolyzing a diverse range of polysaccharides containing β-1,4-linkages; this conclusion is based on the fact that Cel6 CBHs act from non-reducing ends of cellulose (Rouvinen et al. 1990).

Most of the GH6 cellulases are optimally active in the acidic range such as Ctcel6 of *Chaetomium thermophilum* and TlCel6A of *Talaromyces leycettanus* (Zhou et al. 2017; Gu et al. 2019) and also GH7 CBHs of *M. thermophila* (MtCel7A) and *C. thermophilum* (CtCel7) (Kadowaki et al. 2018; Han et al. 2020). Only one CBH (MoCel6A) of *Magnaporthe oryzae* has been reported with the pH optima of 9.0 (Takahashi et al. 2010). It is known that a high pH causes reduction in CBH adsorption to lignin during enzymatic hydrolysis (Lu et al. 2017), thus a high pH optima of rMtCel6A is a beneficial feature for some industrial applications too. The temperature optimum of this recombinant enzyme is 60 °C. Most characterized CBHs have been shown to be active in this temperature range (Table 5). Thermostability studies validate that this enzyme is a thermostable enzyme. Another CBH of this strain (rMtCel7A) is reported to be less thermostable with the T_1/2_ of 60 min at 70 °C as compared to that of rMtCel6A that exhibits prolonged activity at 70 °C (Kadowaki et al. 2018).

**Table 5:** The characteristics of fungal cellobiohydrolases

Protein	GH family	Source organism	Molecular mass (kDa)	Enzyme activity	Optimum pH and Temp. (°C)	Enzyme kinetics	Reference
MtCel6A	6	*Myceliophthora thermophila*	~ 45	172 U mg^**−1**^	10 and 60	K_m_ = 3.2 mg mL ^−1^, V_max_ = 222.2 μM mg ^−1^ min ^−1^	This investigation
TlCel6A	6	*Talaromyces leycettanus*	65	92.9 U mg^**−1**^	5 and 80	NR	Gu et al. 2019
Ctcel6	6	*Chaetomium thermophilum*	42	1.27 U mg^**−1**^	5 and 70	K_m_ = 0.30 mM	Zhou et al. 2017
CelA	6	*Neocallimastix patriciarum* J11	55	200 U mg^**−1**^	6 and 50	NR	Wang et al. 2014
CBH1 and CBH2	NR	*Schizophyllum commune* KMJ820	50 and 150	19.57and 18.12 U mg^**−1**^	5 and 50	K_m_ = 2.0 mM and 1.4 mM V_max_ = 51.4 and 20.8 U mg^−1^	Kondaveeti et al. 2020
CtCel7	7	*Chaetomium thermophilum*	73	1.94 U mg^**−1**^	4 and 60	K_m_ = 4 mg mL^−1^, V_max_ = 18.45 ug min^−1^ mL^−1^ k_cat_/K_m_ = 391 mL s^−1^ mg^−1^	Han et al. 2020
CBHI	7	*Penicillium digitatum*	74	30.9 U mg^**−1**^	5.2 and 60	K_m_ = 11.2 mg mL^−1^, V_max_ = 0.13 μmol min ^−1^, k_cat_/K_m_ = 4.7 (mg mL^−1^)^−1^ s^−1^	Dos Santos et al. 2020
Cbh1	7	*Trichoderma virens* UKM1	54	4.195 U mg^**−1**^	4 and 60	K_m_ = 1.88 mM V_max_ = 41.6 µmole min^−1^ mg^−1^ k_cat_/K_m_ = 5.68 × 10^−4^ mM^−1^ s^−1^	Wahab et al. 2019
Te-Cel7A	7	*Trichoderma reesei*	66	0.93 U L^**−1**^	5 and 65	NR	Sun et al. 2018
MtCel7A	7	*Myceliophthora thermophila*	66	6.8 U mg^**−1**^	4–5 and 70	K_m_ = 0.57 mM, k_cat_/K_m_ = 6.7 M^−1^ s^−1^	Kadowaki et al. 2018
CBH7B	7	*Thielavia terrestris*	51.8	63 U mg^**−1**^	5 and 55	K_m_ = 0.28 mM V_max_ = 402 nmol min^−1^ mg^−1^ k_cat_ = 0.35 s^−1^	Woon et al. 2016

*NR* Not reported

The K_m_ value of this enzyme is lower than those of other reported fungal CBHs (Han et al. 2020; Dos Santos et al. 2020), suggesting that it has a higher substrate affinity and possesses a superior catalytic activity. It is known that Cel6A (CBHII) of *Hypocrea jecorina* is more effective when substrate is abundant, while Cel7A (CBHI) works efficiently when substrate is limiting (Badino et al. 2017). Cel6A is, therefore, an efficient enzyme in cellulose degradation and is able to release a higher amount of cellobiose at much higher rate than Cel7A. Both CBHI and CBHII are produced by cellulolytic fungi in order to efficiently hydrolyze cellulose (Tonozuka et al. 2014).

The enzyme activity of rMtCel6A in the presence of metal ions is comparable with the CBH of *Clostridium clariflavum* and *Trichoderma harzianum*; stimulation in enzyme activity was observed in the presence of Cu^2+^ and Co^2+^ ions (Zafar et al. 2021; Nawaz et al. 2006). The activity of CBH of *Neocallimastix patriciarum* J11 was stimulated by Co^2+^, Mn^2+^ and Cu^2+^ (Wang et al. 2014). Metal ions act as electrophiles and are vital in increasing substrate polarity and the rate of enzyme catalysis by interacting with certain amino acid residues in the active sites of enzymes (Siegel and Siegel 1976). It is evident that rMtCel6A is an organic solvent tolerant cellulase which is a desirable characteristic feature for one-pot production of biofuels. Various industrial processes usually require enzymes to be active in the presence of organic solvents, in which most enzymes often lose their activity (Chapman et al. 2018). Many thermostable enzymes are known to display organic solvent tolerance due to the presence of relatively higher proportion of hydrophobic amino acids (Vieille and Zeikus 2001). rMtCel6A is also a surfactant tolerant enzyme that proves its potential application in the detergent formulations. The stability in the presence of non-ionic surfactants has greater advantages in industrial applications including paper industries. Agrawal et al. (2017) reported that the addition of surfactant during enzymatic hydrolysis is an effective way to improve saccharification.

Previous studies have suggested that an Asp residue is the probable catalytic residue of Cel6A that plays a crucial role in the substrate binding (Rouvinen et al. 1990), which further confirms the observations recorded in this investigation. The reduced activity in presence of WRK, EDAC and NBS predicts the existence of acidic and aromatic amino acid residues and carboxylic groups in the active site of MtCel6A. Trp residues have also been reported to be located at the entrance of the active site tunnel of GH6 CBH of *Phanerochaete chrysosporium* (Yamaguchi et al. 2020). The presence of the Trp residue provides hydrophobic stacking interactions and guide the cellulose chain into the active site tunnel for hydrolytic action (Nakamura et al. 2013). The presence of Asp residues in the catalytic cite of enzyme also confirms that acidic amino acid residues and carboxylic groups are critical in enzyme catalysis. Multiple sequence alignment also validates the significant role of these residues in MtCel6A.

The rMtCel6A also showed salt tolerance with minimal effect of NaCl (upto 2 M) on rMtCel6A activity. The halostability is not reported in other two characterized CBHs of *M. thermophila* (Gusakov et al. 2005; Kadowaki et al. 2018). Thus, this is the first halostable CBH reported in *M. thermophila.*

It is identified that GH6 CBHs are classically exo-type CBHs acting from non-reducing ends (Varrot et al. 1999; Rouvinen et al. 1990). Thus, the end product analysis of hydrolysis utilizing rMtCel6A confirms that this enzyme displays exo-catalytic action with the liberation of cellobiose. The inclusion of rMtCel6A in Cellic CTec2 for hydrolysis suggests that rMtCel6A exerts a synergistic effect leading to enhanced saccharification of the complex cellulosic biomass such as sugarcane bagasse.

This investigation suggests that rMtCel6A exhibits remarkable thermo-alkali stability with detergent and solvent tolerance that makes this CBH useful in various biotechnological processes, where high temperature, alkaline conditions, detergents and solvents prevail. The enzyme activity of rMtCel6A in the presence of a wide range of substrates suggests that this is a processive enzyme with a typical exocellulase activity. The presence of CBHI and CBHII makes the cellulolytic system robust and efficient. The synergistic effect of MtCel6A with commercial cellulases supports its utility in the saccharification of lignocellulosics and bioconversion of cellulosics to bioethanol.

## Supplementary Information

**Additional file 1: Fig. S1.** CD Spectra of rMtCel6A depicting secondary structure of refolded and unfolded IBs.

## Data Availability

All data generated or analyzed during this study are included in this manuscript.
