# Supplementary material for: Recombinant cellobiohydrolase of Myceliophthora thermophila: characterization and applicability in cellulose saccharification
Source: AMB Express. 2021 Nov 4;11:148. doi: 10.1186/s13568-021-01311-8 (PMC8568750; doi:10.1186/s13568-021-01311-8)
Supplement: Supplementary file 1 — Additional file 1: Fig. S1. CD Spectra of rMtCel6A depicting secondary structure of refolded and unfolded IBs. [file 13568_2021_1311_MOESM1_ESM.docx]

**Supplementary Information**

**AMB Express**

**Recombinant Cellobiohydrolase of *Myceliophthora thermophila*: Characterization and Applicability in Cellulose Saccharification**

**Anica Dadwal^a^, Shilpa Sharma^a,b^ and Tulasi Satyanarayana^a,b^***

^a^ Department of Biological Sciences & Engineering, Netaji Subhas Institute of Technology, (University of Delhi), Azad Hind Fauj Marg, Sector-3 Dwarka, New Delhi-110078, India

^b^ Department of Biological Sciences & Engineering, Netaji Subhas University of Technology, Azad Hind Fauj Marg, Sector-3 Dwarka, New Delhi-110078, India

*****Corresponding author: Email: [tsnarayana@gmail.com](mailto:tsnarayana@gmail.com)

**
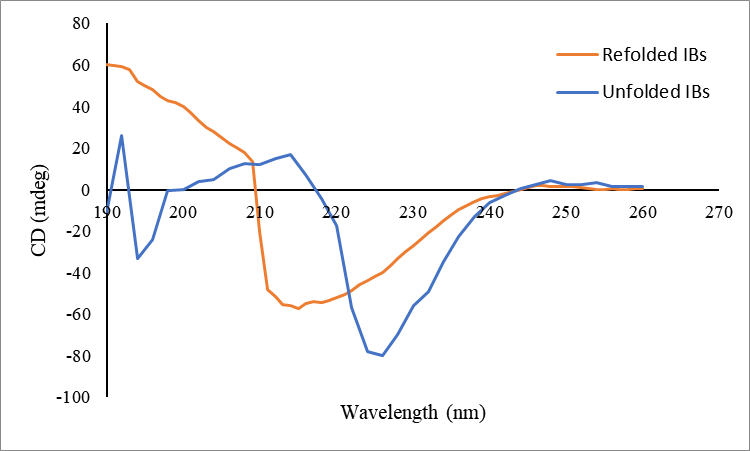
**

**Fig. S1** CD Spectra of rMtCel6A depicting secondary structure of refolded and unfolded IBs.
